# Temperament and character of patients with alcohol toxicity during COVID − 19 pandemic

**DOI:** 10.1186/s12888-021-03052-1

**Published:** 2021-01-20

**Authors:** Ali Reza Estedlal, Arash Mani, Hossein Molavi Vardanjani, Mahsa Kamali, Leila Zarei, Seyed Taghi Heydari, Kamran Bagheri Lankarani

**Affiliations:** 1 Health Policy Research Center, Institute of Heath, Shiraz University of Medical Sciences, Shiraz, Fars Iran; 2 Research Center for Psychiatry and Behavioral Sciences, Shiraz University of Medical Sciences, Shiraz, Iran; 3 MPH Program, School of Medicine, Shiraz University of Medical Sciences, Shiraz, Iran

**Keywords:** COVID-19, Alcohol, Temperament and character, Iran

## Abstract

**Background:**

Corona virus epidemic may be acts as a stressor or trauma that affects both physical health and mental health. People exhibited various reactive behaviors to confront with this stressful situation. In Iran, one of the common motives for alcohol consumption is to scape problems and cope with stresses. It has been shown that personality factors influence alcohol consumption, since they are associated with drinking motives. The main purpose of this study was to investigate the correlation between temperament and character and alcohol abuse.

**Methods:**

This cross-section study was conducted on 135 alcohol intoxicated patients admitted to emergency room in March 2020 and 255 participants who were randomly selected from public in Shiraz. A questionnaire consisted of TCI (Temperament and character inventory) and several questions about COVID-19 pandemic. It was completed by a trained interviewer using the matched answer technique. Demographic factors were self-reported.

**Results:**

Among the alcohol intoxicated group, 117 (86.7%) were males and 18(13.3%) were females. The mean age of the participants was 32.43 ± 10.81 years. Among control group, 99 (38.8%) were males and 156 (61.2%) were females. The mean age of control group was 33.12 ± 14.77 years. Alcohol toxicity was mostly observed among the young males (male/female ratio was 6.45). According to temperament and character index, mean scores of novelty seeking, harm avoidant, and self-transcendence were higher in the alcohol toxicity group than normal population (*P* < 0.01). Mean scores of reward dependent, cooperativeness, and self-directedness were higher in normal population than the alcohol toxicity group (*P* < 0.001). The mean score of persistence was not significantly different between methanol intoxicated and normal population groups (*P* = 0.718).

**Conclusion:**

Alcohol intoxicated patients had higher scores of novelty-seeking and self-transcendence and lower scores of reward-dependency scores, cooperativeness and self-directedness. These scores are associated with higher likelihood of personality disorders.

## Background

COVID-19 (Corona virus disease-2019) Pandemic is an international public health concern. By June 15, 2020, it affected more than 7,274,000 persons, with 413,000 recorded deaths [1]. On 19 February 2020, Iran reported its first confirmed cases of infections in Qom [2]. With rapid increase of the virus at the time of this report on June 15, 2020, Iran was among the top 10 countries reporting COVID-19, with more than 190,000 total confirmed cases [1]. According to a report by Shiraz University of Medical Sciences, since June 10, the COVID-19 disease infected more than 7000 cases in Fars Province, Iran; however, the deaths from the rapidly-spreading disease exceeded 108 patients.

Despite the warning reports on mental health disorders, psychiatric morbidities and behavioral problems during and after similar outbreaks in the past [3], the growing number of COVID-19 cases as well as the unprepared health services have necessitate further efforts to understand the epidemiology, clinical features, transmission patterns, and management of COVID-19 pneumonia [4]. The coincide of these problems with sustained international pressures from economic sanctions have interrupted timely mental healthcare measures [5]; hence, people are more vulnerable to various emotional distress and mental health problems [4, 6].

People exhibited various reactive behaviors to confront with this stressful situation. Some have revealed reasonable behaviors under these conditions; however, a majority of individuals showed maladaptive behavioral responses [7]. Although there are controversies regarding the role of alcohol in removing stress, among these dysfunctional responses, alcohol consumption was reported as one of the most common stress coping methods in this situation in Iran [8]. This has lead to alcohol toxicity epidemic as co-occurring rapidly emerging epidemic besides COVID-19 and thus making syndemics where the one has stimulated the increase of the other [9].

Alcohol production, marketing and consumption are religiously and legally prohibited in Iran and is a punishable crime. Accordingly, bootleg alcohol is available on the black market provided by smuggling and illegal domestic output (under non-standard conditions) [10, 11]. Therefore, in contrast to many countries and cultures that alcohol regularly consumed as a greeting beverage, ritualistic reason or casual leisure activities, alcohol consumption in Iran is very limited and stigmatized. In our experience, a majority of alcohol users in Iran are usually habitual users those who just want to get drunk to scape problems and cope with the stresses. Shortly after the Coronavirus outbreak, different preventive measures were introduced, a majority of which had no scientific basis and were inefficient [12]. For example, with the spread of COVID-19 in Iran, there has been a rumor indicating that drinking or gargling alcohol is effective in preventing or treating the viral pneumonia [13]. Following an official report on the first cases of COVID-19 in Iran (February 26) and with the spread of this rumor, many patients were immediately admitted to the emergency ward, who were intoxicated by bootleg alcohol consumed to prevent this disease. About four weeks later, Iran experienced an outbreak of alcohol toxicity [13]. The startling official stats of more than 3100 methanol toxicity cases and 728 deaths were reported throughout the country from the first official announcement of deaths due to COVID-19 on February 19, 2020 through April 7, 2020 based on Iran’s Health Ministry Spokesman and Iran legal Medicine (LMO) [14]. According to SoltaniNejad et al. [15], these alcohol toxicity outbreaks were reported in 18 (58%) out of 31 provinces in Iran; amongst them, Fars province had the highest level of toxicity [15].

In their Motivational Model of Alcohol Use [16], Cox and Klinger suggested that drinking motives are the most proximal antecedents of alcohol use [16, 17]. According to a large number of studies, the most widely accepted theory to motivate the consumption of alcoholic beverages is underpinned by the role of an interplay between emotional and rational processes, according to which a person makes decisions whether or not to drink alcoholic beverages [18]. These decisions are made based on the affective changes of personal experience, situations, and expectancies [19]. Following previous research addressing the chemical effect of alcohol on the social consequences, there is various motives for drinking alcohol, including social motives videlicet peer acceptance, enhancement motives for drinking to have fun, and coping motives (i.e., tension reduction or distress coping) [17, 19]. Besides, It is evident that other variables such as personality factors evidently influence alcohol consumption as they are associated with drinking motives [20, 21]. As a result, we believe that it is essential to investigate the relationship between personality character and alcohol-related outcomes with regard to the drinking motives [22, 23].

It can be hypothesized that majority of patients who were admitted due to alcohol toxicity consume alcohol for distress coping motive [8, 9]. As we previously mentioned, personality factors influence alcohol consumption, since they are associated with drinking motives [20]. The aim of the study was to investigate and compare the temperament and character of alcohol intoxicated patients and normal population who never had used alcohol in southern of Iran.

## Methods

This cross-section study was conducted on 135 participants in March 2020, who were admitted because of alcohol toxicity to the emergency ward in hospitals affiliated to Shiraz University of Medical Sciences, Shiraz, Iran. Inclusion criteria for the alcohol toxicity group were no history of major psychiatric disorder (such as schizophrenia, major depressive disorder and etc.) and neurologic complaints, at least elementary education, and admission to the emergency room because of alcohol toxicity. Some interviews were conducted with patients just after detoxification when their condition became stable according to the physicians’ report. The patients and their companions, who signed written informed consent, were invited for psychological evaluation. The interviewees were allowed to leave the interviews whenever they wished. The questionnaire was completed by a trained interviewer using the matched answer technique. Demographic factors such as gender, age, level of education, marital status, and economic status were self-reported.

In addition, 255 participants were randomly selected from general public in Shiraz. Due to restrictions on commuting during COVID-19 pandemic, face-to-face interviewing was not possible. Thus, random phone numbers were collected from Shiraz’ phone directory and the questionnaire were completed via phone interviews conducted by the same trained interviewer. The inclusion criteria for the normal population group was not consuming alcoholic beverages, no history of psychiatric and neurologic complications, at least elementary education. The normal population group were asked to submit their oral consent to participate in this study before completing the questionnaires. All the participants completed the questionnaires willingly and were ensured of the confidentiality of the collected data.

All the participants completed the Persian version of the Temperament and Character Inventory-125 (TCI). This scale was developed by Cloninger [24] to evaluate personality traits in seven dimensions. Four dimensions are Novelty seeking (NS), Harm avoidant (HA), reward dependence (RD), and Persistence (PS) (known as “Temperament”), and the other three dimensions are Cooperativeness (CO), Self-directedness (SD), and Self-transcendence (ST) (known as “Character”). The scale was translated into Persian – a native language in Iran – and validated for Iranian population by Kaviani and Poornaseh [25]. In addition, based on public health experts opinions in our research center a yes/no self-report questionnaire were created to address the participants’ beliefs in the beneficial effect of alcohol in preventing and treating COVID-19 and whether they have received news about this subject or not. Moreover, multiple-choice questions about reasons of alcohol consumption (for prevention of COVID-19, habitual consumption or recreational consumption), amount of consumed alcohol (more than once a week, maximum of three times per month and maximum of 6 times per year) and the effect of COVID-19 and the effect of commute restrictions on the alterations of amount of consumption (increase, no change, decrease) were also asked.

The present study was approved by the Ethics Committee of Shiraz University of Medical Sciences (IR.SUMS.REC.1399.313).

### Statistical analysis

Statistical Package for the Social Sciences Version 19.0 (SPSS Inc., Chicago, IL, USA) was used to analyze the data. Frequency (%) and mean ± standard deviation was used as descriptive statistics. Chi-square test was also used to determine the relationship between demographic variables with alcohol toxicity and normal population. Independent sample t-test was conducted to compare the mean scores of temperament and character in alcohol toxicity and normal population groups. Moreover, covariance analysis was performed to adjust gender, age, level of education, marital status, and economic status to further compere compare the mean scores of temperament and character in alcohol toxicity and normal population groups. *P* < 0.05 was considered as the level of significance.

## Results

In this study, the participants were 390 persons (135 persons in alcohol toxicity group and 255 persons in normal population group). Among the alcohol intoxicated group, 117 (86.7%) were males and 18(13.3%) were females. The mean age of the patients was 32.43 ± 10.81 years. Among control group, 99 (38.8%) were males and 156 (61.2%) were females. The mean age of control group was 33.12 ± 14.77 years. Alcohol toxicity was mostly observed among the young males (male/female ratio was 6.45). Moreover, 82 persons (61.2%) hold diploma or had elementary education, and 80 cases (59.3%) reported low economic status (Table 1).

**Table 1:** The relationship between demographic features with alcohol toxicity and normal population

		Normal population	Alcohol Toxicity	*P*-value
Age	< 30	139 (56.3)	58 (43.0)	< 0.001
	30–49	62 (25.1)	65 (48.1)	
	≥50	46 (18.6)	12 (9.1)	
Gender	Male	99 (38.8)	117 (86.7)	< 0.001
	Female	156 (61.2)	18 (13.3)	
Marital Status	Single	151 (59.7)	81 (60.0)	0.952
	Married	102 (40.3)	54 (40.0)	
	Divorced or Widowed	253 (65.2)	135 (34.8)	
Level of education	Elementary	35 (13.9)	30 (22.4)	< 0.001
	Diploma	32 (12.7)	52 (38.8)	
	Bachelor’s	83 (32.9)	50 (37.3)	
	MA or PhD	102 (40.5)	2 (1.5)	
Economic status	Lower income	83 (33.5)	80 (59.3)	< 0.001
	Middle income	108 (43.5)	52 (38.5)	
	High income	57 (23.0)	3 (2.2)	

Among those who were admitted because of alcohol toxicity, 39 cases (28.9%) reported that they had received information about the beneficial effect of alcohol consumption in preventing and treating COVID-19 infection. Among the normal population, 123 participants (49.0%) also have the same self-reports (*P* < 0.001). Among the inpatients, 24 cases (17.9%) believed that alcohol was effective in treating and preventing COVID-19 disease. Similarly, 7 cases (2.8%) of the normal population held the same belief (P < 0.001).

Regarding the reasons for alcohol consumption among those who were admitted because of alcohol toxicity, 20 cases (15%) reported that they used alcohol to prevent COVID-19, 64 cases (48.1%) reported alcohol consumption as a habit, and 49 cases (36.8%) reported recreational consumption of alcohol.

The amount of alcohol consumption in the cases who were admitted due to alcohol toxicity varied. In this regard, 82 (60.7%) cases reported that they used to drink alcoholic beverages more than once a week, 31 (23.0%) cases reported they drink at most three times of drinking per month, and 22 cases (16.3%) reported at most six times of drinking per year. However, 35 cases (26.5%) has increased their consumption during COVID pandemic, 90 cases (68.1%) reported no significant change in their consumption, and 7 cases (5.3%) reported decreased consumption.

According to independent sample t-tests, the mean scores of novelty seeking, harm avoidant, and self-transcendent were higher in the alcohol toxicity group than normal population (*P* < 0.01). In contrast, the mean scores of reward dependent, cooperativeness, and self-directedness were higher in normal population than the alcohol toxicity group (*P* < 0.001). The mean score of persistence was not significant in both group (*P* = 0.718). (Table 1).

To adjust for gender, age, level of education, marital status, and economic status based on covariance analysis, the mean scores of novelty seeking and self-transcendent were higher in the alcohol toxicity group than normal population (*P* < 0.001). The mean scores of reward dependent, cooperativeness, and self-directedness were also higher in normal population than the alcohol toxicity group (P < 0.001). The mean scores of persistent and harm avoidant were not significant in both group (*P* > 0.05). (Table 2).

**Table 2:** Relationship of temperament and character items between alcohol toxicity and normal population

		Number	Mean	Std. Deviation	*P* value**	P value***
Novelty seeking	Normal population	255	8.21	3.34	< 0.001	< 0.001
	Alcohol toxicity	135	10.27	2.06		
Harm Avoidant	Normal population	255	7.86	4.60	0.002	0.203
	Alcohol toxicity	135	8.93	2.31		
Reward Dependent	Normal population	255	8.38	2.43	< 0.001	< 0.001
	Alcohol toxicity	135	6.65	1.77		
Persistence	Normal population	255	3.18	1.42	0.718	0.448
	Alcohol toxicity	135	3.22	1.05		
Cooperativeness	Normal population	255	17.68	3.84	< 0.001	< 0.001
	Alcohol toxicity	135	11.59	2.76		
Self-directedness	Normal population	255	14.64	5.19	< 0.001	< 0.001
	Alcohol toxicity	135	8.21	3.43		
Self-transcendent	Normal population	255	9.16	3.17	< 0.001	< 0.001
	Alcohol toxicity	135	10.75	2.78		

** Based on independent t-test

*** Analysis of covariance adjusted by gender, age, level of education, marital status, economic status

## Discussion

This study has investigated the temperament and character of alcohol intoxicated patients in comparison with population who never had used alcohol. Among temperament dimensions, novelty-seeking and self-transcendence scores were higher and reward-dependency score was lower among the alcohol abusers. However, among character dimensions, cooperativeness and self-directedness scores were lower among the alcohol abusers. These findings are consistent with most of the similar studies [23, 26–29]. In this study the alcohol abusers were mainly males, aged below 30 years, and had lower levels of education and lower economic status. This findings is compatible with previous studies [11]. Some fundamental works in this field also noted that drinking for coping motives is more prevalent among younger male especially after adolescence [18]. This lends support to the assumption that alcohol toxicity population of our study responded to the stressful situation by consuming alcohol. Besides, the findings of the interviews in this study showed that almost half of the normal population and one third of alcohol intoxicated patients had heard the rumor that alcohol consumption has beneficial effect in prevention or treatment of COVID-19. 15% of the alcohol intoxicated patients declared that they consumed alcohol to prevent the disease. On the other hand, a majority of abusers declared that they consume alcohol excessively and habitually (more than once per week).

Preliminary works in this field reported TCI character dimensions to be moderately and homogeneously related to nearly all the personality disorders [30–32]. Lower self-directedness and lower cooperativeness indicate the higher likelihood of personality disorder, according to Cloninger’s hypothesis and many other studies [30, 32]. Lower self-directedness is associated with difficulty accepting responsibility, setting meaningful goals, resourcefully meeting challenges, accepting limitations and disciplining habits to maintain the harmony with their goals and values. Besides, individuals with lower cooperativeness are excessively self-centered, socially intolerant and unhelpful to others lacking empathy, compassion or principles [32]. These are assumed as the core features of personality disorders [32]. In addition, several studies claimed that cluster A personality disorder symptoms (in particular, schizoid personality disorder symptoms) are correlated with low reward-dependence scores. Furthermore, Higher novelty-seeking was correlated with higher impulsiveness, exploratory excitability, extravagance, and disorderliness, which are included in Cluster B personality disorders and associated with alcohol and drug involvement [30, 33].

Body of literature has investigated in order to find correlation between TCI and alcohol abuse. Our experiments are in line with previous results. As expected, higher scores of novelty-seeking, lower scores of self-directedness and lower scores of cooperativeness were the most relevant traits in alcohol and other substance abuse [26–29, 34, 35]. Moreover, based on references, lower reward-dependence score is a highly reported score among alcohol abusers [36–38]. In this study, self-transcendence showed inverse relationship with alcohol consumption. In one study of temperament and character of heroin and alcohol abusers by Le Bon et al. [29] in spite of higher scores of novelty seeking and lower scores of self-directedness, self-transcendence score was also higher in alcohol abusers and more significantly heroin abusers groups in comparison to normal population. However, multiple studies support the protective role of spirituality and self-transcendence in vulnerability to alcohol consumption that demonstrates the protective role of human beings’ internal development to maintain conducts that contribute to states of well-being and health [39–41].

In addition, a review of the literature on harm avoidance documented inconsistent findings among alcohol abuser’s populations [28, 42, 43]. However, the results were not statistically significant in the present study.

## Conclusion

We believe that having a perception of personality traits would be beneficial for clinical practice of mental health professionals. The psychobiological theory identifies seven personality dimensions form both the biologic (temperament) aspect and behavioral (character) aspect. In this study, specific temperamental and character personality dimensions are introduced for alcohol intoxicated patients. Among temperament dimensions, higher novelty-seeking and self-transcendence scores and lower reward-dependency score were indicative of alcohol abuse. Besides, among character dimensions, lower cooperativeness and self-directedness scores were more prevalent among the alcohol abusers. These dimensions of temperament and character have correlation with several personality disorders such as Cluster B personality disorder symptoms (impulsiveness, exploratory excitability, extravagance, and disorderliness) and Cluster A personality disorder symptoms (in particular, schizoid personality disorder symptoms).

### Limitations of the study

We aware that our research may have several limitations. The first is that since we interviewed the alcohol toxicity cases, who were admitted in emergency ward, a majority of the interviewees had decreased level of consciousness, hence it was inevitable to exclude them from the study. Second, this study was conducted during March 2020 in the first days of COVID-19 outbreak in Iran. Consequently, it was not possible and more importantly was not ethical to have face-to-face interview with the control group. We have randomly selected the telephone numbers and had phone interview with the control group. While our cases were mostly men, women and younger individuals were more willing to participate phone interview for control group. However, fortunately, due to our considerable number of participants (390) it was possible to adjust the results for age and gender and use parametric analysis. Third, suspect to measurement bias, owing to the fact that cases were face to face interviewed but controls were phone interviewed Cases might be more reductant to answer than controls. Forth, as a consequence of the religious and legal prohibition of alcohol consumption and the existing stigma towards this subject, the published data on alcohol abuse and its epidemiology are highly limited in Iran; thus, the comparative analysis of the findings was not utterly feasible.

## Data Availability

The datasets used and/or analyzed during the current study available from the corresponding author on reasonable request.

## Abbreviations

TCI: Temperament and character inventory
OR: Odds ratio
NS: Novelty seeking
HA: Harm avoidant
RD: Reward dependence
PS: Persistence
CO: Cooperativeness
SD: Self-directedness
ST: Self-transcendence
